# The association between physical activity and depressive symptoms in adolescents: the serial mediating roles of school connectedness and self-control

**DOI:** 10.3389/fpubh.2026.1872650

**Published:** 2026-07-08

**Authors:** Zhengying Han, Tao Xu

**Affiliations:** College of Physical Education and Health, Guangxi Normal University, Guilin, China

**Keywords:** adolescent, depressive symptoms, physical activity, school connectedness, self-control

## Abstract

**Objective:**

This study examined the association between physical activity (PA) and depressive symptoms (DP) among adolescents, with a particular focus on whether school connectedness (SCH) and self-control (SCT) served as sequential indirect pathways in this association.

**Methods:**

Data were collected from 1,439 adolescents using established questionnaires assessing PA, DP, SCH, and SCT. The dataset was analyzed with SPSS 29.0 and AMOS 27.0.

**Results:**

PA was inversely associated with DP (*r* = −0.330, *p* < 0.001). Further analyses showed that SCH and SCT both carried part of the effect of PA on DP. The indirect effect through SCH was −0.0423, whereas the indirect effect through SCT was −0.0069. A significant serial pathway from PA to SCH, then to SCT, and finally to DP was also observed, with an effect size of −0.0158. The combined indirect effect was −0.065, explaining 86.67% of the total effect.

**Conclusion:**

Higher levels of PA were associated with fewer DP among adolescents. After SCH and SCT were included in the model, the direct association between PA and DP was no longer statistically significant, suggesting that SCH and SCT may play important indirect roles in the PA–DP relationship. Given the cross-sectional design, these findings should be interpreted as statistical associations rather than evidence of causal mechanisms.

## 1 Introduction

Depressive Symptoms (DP) describe a set of persistent negative emotional experiences, typically reflected in low mood and decreased engagement in usual activities, often together with fatigue, altered sleep or appetite, impaired attention, and a more negative view of oneself (1). DP in adolescence have become a major global public health concern. Current evidence indicates that the overall prevalence of adolescent DP worldwide is approximately 20.5% (2), whereas the prevalence among Chinese adolescents reaches 26.17% (3), exceeding the global average and highlighting the urgency of this issue in China. From the perspective of developmental psychopathology, adolescence represents a sensitive period for the emergence, accumulation, and manifestation of depressive problems. The risk of depression rises substantially after puberty, and DP during this stage are closely associated with subsequent social dysfunction, the persistence of affective disorders, and poor mental health outcomes in adulthood (4). Among the wide range of adolescent mental health problems, DP are among the most common and clinically significant internalizing difficulties. In addition to being highly prevalent during adolescence, they are strongly linked to impaired academic functioning, maladaptive social adjustment, and long-term mental health risk (5). Moreover, DP rarely occur in isolation; rather, they are often intertwined with anxiety, stress, and school-related factors, showing considerable comorbidity and complex interactions (6). Therefore, identifying the factors associated with adolescent DP and clarifying their underlying mechanisms are essential for promoting youth mental health and developing timely preventive interventions.

Physical activity (PA) refers broadly to intentional and repeated movement that contributes to better physical functioning (7). However, national data from China suggest that only 29.9–34.1% of children and adolescents reach the advised daily duration of moderate-to-vigorous activity, highlighting a widespread lack of adequate PA in this population. This insufficiency is particularly pronounced among girls and older students (8). Accordingly, insufficient PA among adolescents has become an important public health issue. From a biopsychosocial perspective, adolescent DP do not arise from a single cause but instead reflect the combined influence of physiological regulation, psychological adaptation, and environmental factors. Previous studies have suggested that PA may be associated with lower levels of DP not only through biological pathways—such as the regulation of neurotransmitters, neurotrophic factors, and inflammatory responses—but also through improvements in emotional state and psychological functioning (9). Empirical evidence further suggests that PA can alleviate depressive symptoms to some extent, with moderate-to-vigorous activity showing particularly favorable effects (Pérez (10)). Aerobic exercise has also been shown to significantly normalize amino acid metabolites in the hippocampus, thereby contributing to improvements in depressive states (11). More broadly, PA has been reported to promote both cognitive functioning and mental health among healthy adolescents as well as those experiencing psychological difficulties (12). Compared with many other intervention approaches, PA is relatively safe, accessible, and cost-effective, and may help avoid some of the physiological side effects associated with pharmacological treatment. Against the backdrop of generally insufficient PA among Chinese adolescents, examining the relationship between PA and DP is therefore of considerable theoretical and practical significance. Based on the above evidence, we proposed H1: adolescents with higher levels of PA would be expected to report lower levels of DP.

School Connectedness (SCH) reflects how strongly students feel connected to their school environment, including whether they perceive understanding, recognition, and emotional closeness from teachers and classmates (13). A growing body of research suggests that SCH is closely related not only to students’ learning involvement, conduct, and adjustment at school, but also to broader indicators of physical and psychological health. Higher levels of SCH generally indicate that students experience greater support, acceptance, and belonging within the school environment, which in turn may reduce emotional distress and lower the risk of DP (14). From the perspective of stage–environment fit theory, SCH is particularly important during adolescence. This developmental period is characterized by rapid changes in self-identity, peer relationships, and social adjustment, and adolescents’ emotional adaptation may depend partly on whether the school environment satisfies their needs for belonging, recognition, and support (15). Physical activity (PA), especially physical education, extracurricular sports, and team-based activities in school settings, may provide adolescents with more opportunities for peer cooperation, teacher–student interaction, and collective participation. Through these experiences, students may develop positive activity-related experiences and perceive stronger support from peers and teachers, thereby strengthening their sense of belonging, identification, and emotional connection with school. Therefore, SCH can be regarded as an important school-based contextual resource that may help explain the association between PA and DP among adolescents (16). A longitudinal study has shown that SCH serves a protective role against DP, such that higher SCH predicts lower subsequent levels of DP (17). Meta-analytic evidence further shows that SCH is closely linked to better mental health in adolescents. In particular, more supportive teacher–student interactions and a stronger sense of attachment to school tend to coincide with lower levels of depressive symptoms (18). Taken together, these results imply that SCH may serve as an important protective resource in relation to adolescent DP. As an important health-related behavior situated within the school context, PA may also contribute to the development of SCH. Whether through regular physical education classes, extracurricular exercise, or team-based sports participation, PA provides adolescents with more opportunities for peer cooperation, teacher interaction, and integration into the collective, thereby strengthening their sense of school identification, support, and belonging (19). Previous studies have shown that greater opportunities for PA participation are associated with stronger feelings of social closeness at school, while adolescents with higher SCH also tend to report higher levels of PA participation (20, 21). Taken together, these findings suggest that PA may be indirectly associated with DP through SCH. Accordingly, we proposed H2: SCH mediates the association between PA and DP.

Self-Control (SCT) refers to the ability to control impulses and stay focused on intended goals. This process often consumes psychological resources such as energy and willpower (22). The strength model proposes that self-regulation draws on limited psychological resources; thus, higher SCT may help individuals deal more effectively with both inner impulses and outside interference (23). A meta-analysis covering 48 studies and 75,541 participants showed that PA was positively related to SCT, and the overall effect was in the small-to-moderate range (ES = 0.213) (24). In addition, higher PA has been linked to fewer problematic outcomes, which suggests that SCT may be an important pathway through which PA supports psychological well-being and lowers the likelihood of risky behavior (25). SCT was included in the present model because it reflects adolescents’ internal capacity for self-regulation. PA is not merely a form of bodily movement; it often involves rule adherence, goal persistence, delayed gratification, fatigue tolerance, and impulse inhibition. These behavioral demands are closely related to the development of SCT. Adolescents who regularly engage in PA may gradually develop stronger behavioral regulation and goal-management skills through sustained participation. Therefore, SCT can be considered an important internal psychological factor that may help explain the association between PA and DP among adolescents. On the one hand, PA may be associated with SCT through improvements in brain function and neural regulation. Exercise has been linked to enhanced neurotrophic support, cerebral blood flow, and neuroplasticity, all of which may strengthen higher-order cognitive processes governed by the prefrontal cortex; inhibitory control, in particular, is a core component of prefrontal functioning (26). Thus, PA is not merely a behavioral activity but may also enhance individuals’ ability to regulate impulses and resist distraction through neurophysiological pathways. On the other hand, SCT is closely related to DP. Existing evidence suggests that adolescents with stronger SCT generally report fewer DP, and this pattern has also been confirmed in longitudinal research (24). In addition, a systematic review of self-regulation-based interventions in youth found that enhancing SCT not only improves behavior and academic functioning but may also alleviate depressive problems (27). This may mean that adolescents with lower SCT have more difficulty managing negative emotions and dealing with stress, which could place them at greater risk for DP. Taken together, these findings imply that PA may be related to lower DP in part through its association with SCT. On this basis, H3 stated that SCT would function as an indirect pathway linking PA to DP.

PA may not only enhance SCT but also strengthen SCH, suggesting that these two variables may jointly constitute important pathways through which PA influences adolescent mental health. From the perspective of self-determination theory, participation in PA may help adolescents fulfill key developmental needs, including feeling capable, connected to others, and able to act with a sense of choice. This process may not only promote sustained intrinsic motivation for exercise but also facilitate more positive school experiences and stronger self-regulatory capacity (28). Specifically, regular participation in PA provides adolescents with increased opportunities for peer cooperation, teacher interaction, and collective involvement, all of which may enhance SCH. At the same time, the demands inherent in PA—such as goal persistence, rule adherence, and delayed gratification—may further promote the development of SCT. There is also a close internal connection between SCH and SCT (29). From an ecological systems perspective, the growth of SCT in adolescence is influenced by the surrounding environment over time. Among these contexts, school is especially important because it helps shape behavioral standards and supports the development of self-regulatory abilities (30). Higher SCH implies that students receive more positive interactions and developmental support in the school setting. This may strengthen their identification with school rules and collective goals, thereby fostering more stable behavioral regulation and goal-directed functioning, which in turn facilitates the development of SCT, including impulse control, resistance to temptation, sustained task focus, and the maintenance of healthy habits (31). Importantly, both SCH and SCT have been identified as protective factors against adolescent DP. Stronger SCH allows adolescents to experience greater support, recognition, and belonging, and may be linked to lower emotional distress. Likewise, stronger SCT enhances the ability to regulate impulses, negative emotions, and stress responses, may be associated with lower DP (32). Taken together, PA may be associated with adolescent DP not only directly but also through indirect pathways involving SCH and SCT. Accordingly, H4 proposed that SCH and SCT would form a sequential indirect pathway linking PA to DP.

**Figure 1:**
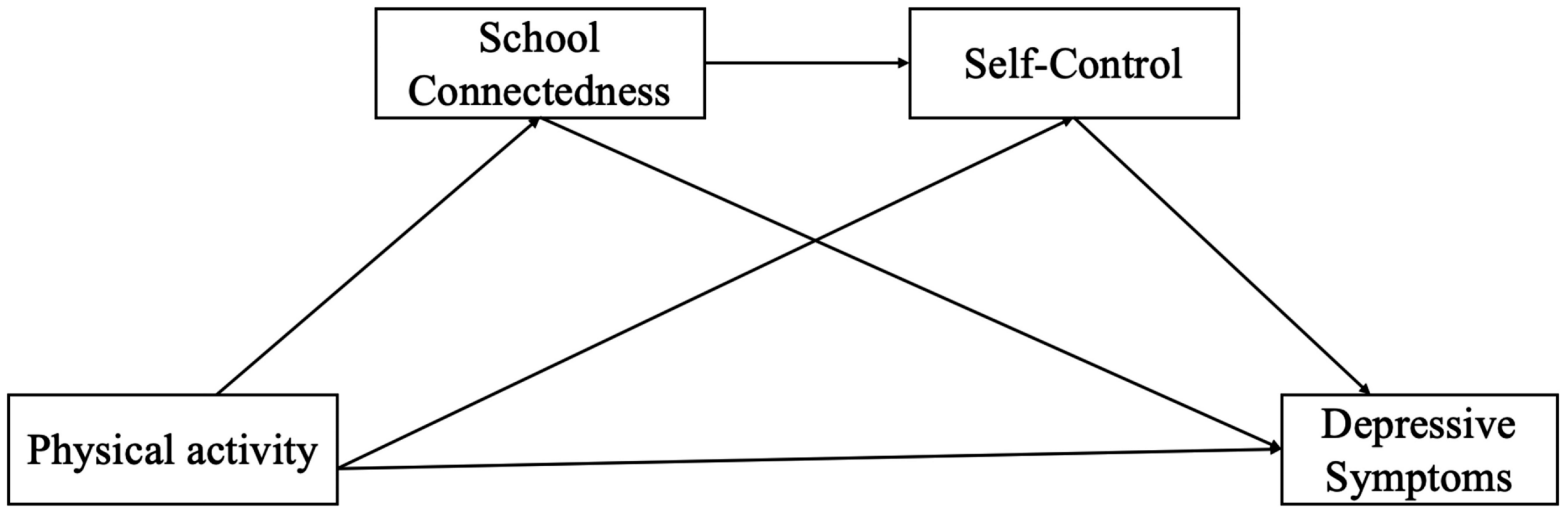
Serial mediation model.

Although previous studies have shown that adolescents with higher levels of PA tend to report fewer DP, the psychosocial pathways underlying this association remain insufficiently clarified. Existing research has mainly examined the direct link between PA and adolescent mental health, while giving less attention to school-based contextual resources and individual self-regulatory processes. In particular, SCH and SCT have both been regarded as important protective factors for adolescent DP, yet they have often been investigated separately rather than as components of a shared explanatory pathway. SCH reflects the external support, belonging, and recognition that adolescents experience within the school environment, whereas SCT represents their internal capacity to regulate impulses, sustain goals, and manage behavior. Examining SCH and SCT simultaneously may therefore offer a more integrated understanding of the PA–DP association from an “external school support–internal self-regulation” perspective. Accordingly, the present study proposed a serial mediation model in which PA is indirectly associated with DP through SCH and SCT, aiming to test a theoretically grounded indirect pathway linking PA to adolescent DP (Figure 1). Based on this rationale, four hypotheses were developed:

*H1*: Higher PA would be related to fewer DP.

*H2*: SCH would act as an indirect pathway between PA and DP.

*H3*: SCT would also serve as an indirect pathway between PA and DP.

*H4*: SCH and SCT would jointly form a sequential pathway linking PA to DP.

## 2 Methods

### 2.1 Participants

This study was conducted in March 2026 in six cooperating schools in the Guangxi Zhuang Autonomous Region, China. Convenience sampling was used. The participating schools were selected according to the accessibility of the research team, schools’ willingness to cooperate, and the feasibility of on-site data collection. With the assistance of school administrators and head teachers, eligible students completed the questionnaires by class. The study was approved by the Ethics Committee of Guangxi Normal University (Approval No. 20251230001). All participating students and their legal guardians provided voluntary informed consent after being informed of the study purpose, survey content, and confidentiality procedures. Students were eligible if they were enrolled in junior or senior high school, were able to understand the questionnaire and complete it independently, agreed to take part in the study, and provided complete data on the main variables. Students were excluded if they had severe physical illness, neurological disorders, or psychiatric conditions that could interfere with participation, were absent on the day of the survey, or submitted questionnaires with substantial missing information or clearly invalid response patterns. A total of 1,700 questionnaires were distributed, and 1,439 were retained for analysis, resulting in a valid response rate of 84.65%. The final sample consisted of 772 boys (53.65%) and 667 girls (46.35%), with an average age of 14.35 ± 1.93 years. Of these participants, 1,227 were junior high school students (85.27%) and 212 were senior high school students (14.73%). In addition, 944 students were from urban areas (65.60%), whereas 495 were from rural areas (34.40%).

### 2.2 Measures

#### 2.2.1 PA

PA was measured with Liang Deqing’s revised PA Rating Scale (33). This instrument contains three questions covering exercise intensity, time, and frequency, with each item scored on a five-point response scale. The overall score was derived by multiplying intensity × (time − 1) × frequency, producing values from 0 to 100. Larger values represent greater participation in PA. In this sample, the scale showed acceptable reliability, with a Cronbach’s *α* of 0.742 and a 14-day retest coefficient of 0.780 (*p* < 0.05).

#### 2.2.2 DP

DP were assessed with the Patient Health Questionnaire-9 (1). Earlier studies have supported the psychometric adequacy of the Chinese PHQ-9, including its use with adolescent samples (34, 35). This measure includes nine questions, scored from 0 to 3, where 0 indicates “not at all” and 3 indicates “nearly every day.” The summed score spans 0–27, and larger totals reflect greater severity of DP. In the current sample, the instrument showed strong reliability, with Cronbach’s *α* = 0.893 and a 14-day retest coefficient of 0.780 (*p* < 0.05). The CFA results also supported acceptable structural validity, with *χ^2^*/df = 3.034, GFI = 0.899, TLI = 0.910, and RMSEA = 0.065.

#### 2.2.3 SCH

SCH was measured with the School Connectedness Scale developed by Yu Chengfu and colleagues (36). The instrument contains 10 statements; each answered on a five-point scale from 1 (“strongly disagree”) to 5 (“strongly agree”). Possible total scores extend from 10 to 50, and higher values indicate a stronger sense of connection to school. In this study, the measure showed acceptable reliability, with Cronbach’s *α* = 0.722 and a 14-day retest coefficient of 0.792 (*p* < 0.05). Its structural validity was also supported by the CFA results, which showed an acceptable fit: *χ^2^*/df = 3.063, GFI = 0.869, TLI = 0.898, and RMSEA = 0.050.

#### 2.2.4 SCT

SCT was evaluated with the Self-Control Scale developed by Tangney et al. (37) and subsequently adapted for Chinese participants by Tan and Guo (38). This instrument contains 19 items, each scored on a five-point response format, with options ranging from 1 (“completely inconsistent”) to 5 (“completely consistent”). The summed score varies between 19 and 95, and a larger total reflects better SCT. In the present sample, the measure showed good reliability, with Cronbach’s α = 0.819 and a 14-day retest coefficient of 0.882 (*p* < 0.05). The CFA results further supported acceptable construct validity, with *χ^2^*/df = 3.790, GFI = 0.921, TLI = 0.917, and RMSEA = 0.056.

### 2.3 Covariates

Previous studies have suggested that demographic characteristics, including gender, school stage, urban–rural residence, and parental educational attainment, may influence adolescents’ PA, SCH, SCT, and DP, thereby potentially confounding the associations among the focal variables. To minimize potential confounding and clarify how PA relates to DP, together with the sequential pathway involving SCH and SCT, these demographic characteristics were controlled for in the statistical analyses.

### 2.4 Statistical analysis

AMOS 27.0 was used to conduct confirmatory factor analysis. Prior to the main analyses, all variables were standardized. Common method bias was assessed using both Harman’s one-factor test in SPSS 29.0 and a supplementary single-factor confirmatory factor analysis, in which all core items were loaded onto one latent factor. Pearson correlation analyses were then performed to examine the associations among PA, DP, SCT, and SCH. The hypothesized sequential mediation model was tested using PROCESS Model 6 in SPSS (39). Indirect effects were estimated based on 5,000 bootstrap samples, and mediation effects were considered statistically significant when the 95% confidence interval did not include zero. The significance level was set at *α* = 0.05, Table 1.

**Table 1:** Demographic characteristics of the participants (*N* = 1,439).

Characteristic	Category	n	%
Gender	Boys	772	53.65
	Girls	667	46.35
School stage	Junior high school	1,227	85.27
	Senior high school	212	14.73
Residence	Rural	495	34.40
	Urban	944	65.60
Mother’s education	Primary school or below	487	33.84
	Secondary school	844	58.65
	College or above	108	7.51
Father’s education	Primary school or below	287	19.95
	Secondary school	1,017	70.67
	College or above	135	9.38

## 3 Results

### 3.1 Common method bias

Common method bias was first assessed using Harman’s one-factor test, following the recommendations of Podsakoff et al. (40). The unrotated exploratory factor analysis extracted four factors with eigenvalues greater than 1. The first factor accounted for 24.23% of the total variance, which was below the commonly used 40% threshold. As a supplementary check, a single-factor confirmatory factor analysis was conducted by loading all core items onto one latent factor. The results indicated poor model fit: *χ^2^*/df = 12.755, CFI = 0.633, TLI = 0.614, RMSEA = 0.090, and SRMR = 0.088. These findings suggest that a single common factor was unlikely to explain the covariance among the study items, indicating that common method bias was unlikely to seriously distort the main findings. Nevertheless, because all core variables were measured using self-report questionnaires at the same time point, the possibility of common method bias cannot be completely ruled out, and the results should therefore be interpreted with caution.

### 3.2 Differences across demographic groups

Table 2 indicates that the major study variables varied across several demographic groups. In the gender comparison, boys tended to report higher PA, SCH, and SCT, whereas girls showed higher DP. A similar pattern emerged across school stages: junior high school students generally scored higher on PA, SCH, and SCT, while senior high school students reported more DP. Urban students also performed better than rural students on PA, SCH, and SCT, whereas rural students showed comparatively higher DP. In addition, differences related to paternal educational level were found for PA, SCH, and SCT. Overall, these findings suggest that demographic characteristics should be considered when examining the links among PA, SCH, SCT, and DP.

**Table 2:** Differences in PA, SCH, SCT, and DP across demographic characteristics (M ± SD).

Variable		PA	SCH	SCT	DP
Gender	Boys	39.75 ± 24.68	36.98 ± 8.40	43.40 ± 14.10	7.51 ± 5.49
	Girls	24.24 ± 18.62	34.19 ± 8.70	40.19 ± 14.84	9.69 ± 6.15
	T	13.553	6.161	4.201	−7.076
	P	<0.001	<0.001	<0.001	<0.001
School stage	Junior high school	33.75 ± 23.08	36.23 ± 8.75	42.74 ± 14.89	8.38 ± 6.01
	Senior high school	25.69 ± 24.04	32.58 ± 7.38	37.20 ± 11.19	9.37 ± 5.20
	T	4.534	6.450	6.301	−2.496
	P	<0.001	<0.001	<0.001	0.013
Residence	Urban	35.09 ± 19.71	37.91 ± 9.07	47.71 ± 14.74	7.51 ± 5.33
	Rural	31.23 ± 25.02	34.52 ± 8.19	38.89 ± 13.47	9.06 ± 6.12
	T	3.204	6.954	11.102	−4.989
	P	<0.001	<0.001	<0.001	<0.001
Mother’s education	Primary school or below	31.61 ± 21.91	35.65 ± 9.21	42.84 ± 14.51	8.72 ± 5.93
	Secondary school	32.61 ± 23.41	35.77 ± 8.19	41.93 ± 13.97	8.21 ± 5.51
	College or above	37.81 ± 25.61	35.73 ± 9.17	41.02 ± 15.10	9.16 ± 6.71
	F	2.350	0.132	1.394	2.329
	P	0.052	0.971	0.234	0.054
Father’s education	Primary school or below	21.65 ± 17.12	35.44 ± 8.99	38.69 ± 14.80	8.87 ± 5.84
	Secondary school	33.68 ± 22.88	36.09 ± 8.68	42.28 ± 15.14	8.19 ± 5.65
	College or above	35.68 ± 26.67	36.42 ± 8.61	42.93 ± 13.94	9.23 ± 6.39
	F	3.364	2.637	4.463	2.241
	P	0.009	0.033	0.001	0.063

Table 3 shows a clear overall pattern among the core variables. PA was positively related to both SCH and SCT, while showing an inverse relationship with DP. SCH was likewise positively linked to SCT and negatively linked to DP. In addition, higher SCT was accompanied by lower DP. Taken together, these findings indicate that adolescents with greater PA, stronger school connection, and better self-regulation tended to report fewer depressive symptoms.

**Table 3:** Correlations between variables and factors.

Variable	M ± SD	PA	SCH	SCT	DP
PA	32.56 ± 23.39	—			
SCH	35.69 ± 8.65	0.435***	—		
SCT	41.94 ± 14.53	0.302***	0.536***	—	
DP	8.52 ± 5.90	−0.330***	−0.598***	−0.550***	—

^***^p<0.001.

According to Table 4, after controlling for gender, school stage, residence, and parental education, PA was positively associated with both SCH and SCT. In addition, SCH showed a significant positive association with SCT. These results were consistent with the proposed serial mediation model, suggesting that SCH and SCT may be involved in the indirect association between PA and DP. However, because the data were cross-sectional, this pathway should be interpreted as an exploratory statistical association rather than evidence of a causal process.

**Table 4:** Results of the serial mediation analysis.

Model	Predictor	Outcome	*R*	*R* ^2^	*F*	*β*	95% CI		*t*	*p*
							95% CI Lower	95% CI Upper		
Model1		SCH	0.466	0.217	66.230					
	Gender					−0.267	−1.111	0.578	−0.619	0.536
	School stage					−1.190	−2.418	0.038	−1.900	0.058
	Residence					−2.771	−3.752	−1.789	−5.538	<0.001
	Father’s education					0.355	−0.210	0.920	1.232	0.218
	Mother’s education					0.024	−0.540	0.587	0.082	0.935
	PA					0.152	0.134	0.170	16.479	<0.001
Model 2		SCT	0.575	0.331	101.040					
	Gender					−0.075	−1.387	1.237	−0.112	0.911
	School stage					0.357	−1.553	2.267	0.367	0.714
	Residence					−6.296	−7.837	−4.756	−8.017	<0.001
	Father’s education					0.238	−0.641	1.116	0.531	0.596
	Mother’s education					0.115	−0.760	0.991	0.258	0.796
	PA					0.052	0.021	0.083	3.321	0.001
	SCH					0.777	0.696	0.857	18.924	<0.001
Model 3		DP	0.666	0.443	142.447					
	Gender					0.870	0.338	1.356	3.509	<0.001
	School stage					−0.775	−1.483	−0.067	−2.147	0.032
	Residence					−0.425	−1.009	0.158	−1.430	0.153
	Father’s education					−0.007	−0.333	0.318	−0.045	0.964
	Mother’s education					−0.030	−0.354	0.295	−0.179	0.858
	PA					−0.010	−0.021	0.002	−1.635	0.102
	SCH					−0.278	−0.311	−0.244	−16.333	<0.001
	SCT					−0.134	−0.153	−0.114	−13.632	<0.001

**Figure 2:**
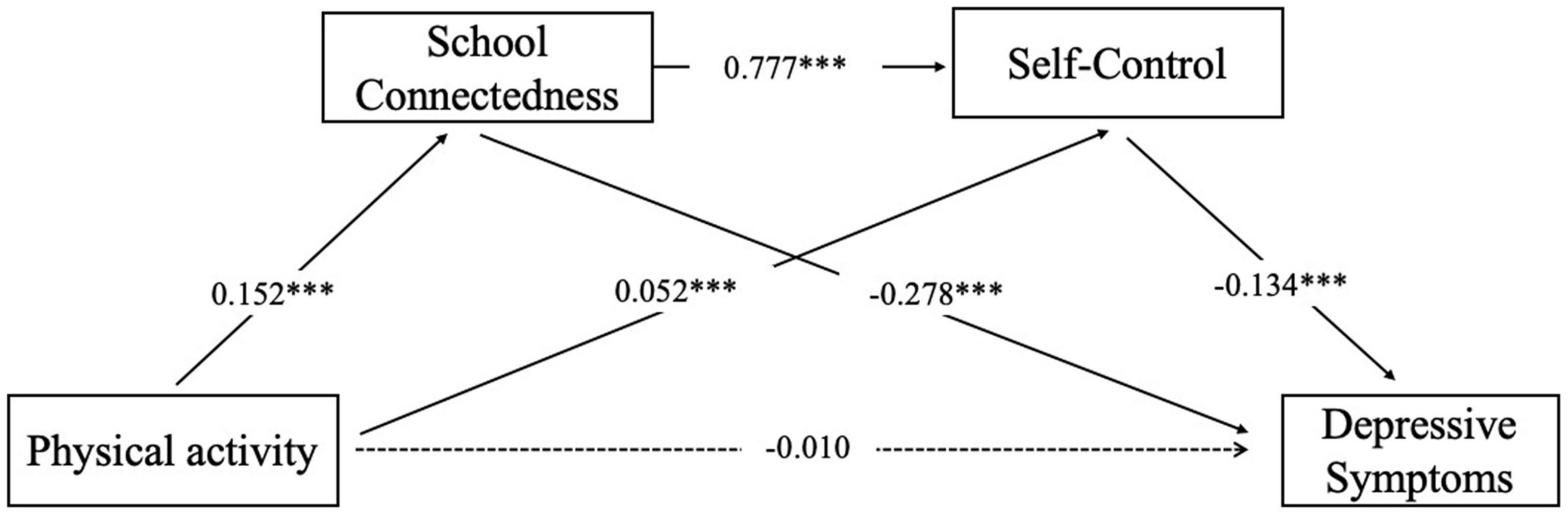
Bootstrap analysis of the significance and effect sizes of the mediation effects.

According to Table 5 and Figure 2, the indirect effect of PA on DP through SCH accounted for 56.01% of the total effect, whereas the indirect effect through SCT accounted for 9.33%. In addition, the serial indirect pathway through SCH and SCT accounted for 21.33% of the total effect. The 95% confidence intervals for all three indirect pathways did not include zero, indicating that these indirect effects were statistically significant. Overall, the pathway involving SCH contributed the largest relative share among the three indirect pathways, while the independent pathway through SCT showed a smaller contribution. These findings suggest that different indirect pathways may play unequal roles in explaining the association between PA and DP. Given the cross-sectional design, these results should be interpreted as exploratory statistical associations rather than evidence of causal transmission.

**Table 5:** Path coefficients and effect decomposition.

Effect	Path	Effect size (95% CI)	Proportion (%)
Total effect	—	−0.075(−0.088, −0.062)	100.00
Direct effect	PA → DP	−0.010 (−0.021, 0.002)	13.33
Total indirect effect	—	−0.065(−0.074, −0.055)	86.67
Indirect effect 1	PA → SCH → DP	−0.042(−0.051, −0.034)	56.01
Indirect effect 2	PA → SCT → DP	−0.007(−0.012, −0.003)	9.33
Indirect effect 3	PA → SCH → SCT → DP	−0.016(−0.020, −0.013)	21.33

## 4 Discussion

### 4.1 PA was significantly negatively associated with DP

The results indicated that adolescents with higher levels of PA tended to report fewer DP, supporting H1 (41). This finding is in line with behavioral activation theory, which proposes that sustained, goal-directed engagement in positive activities increases exposure to positive reinforcement while reducing avoidance and withdrawal, thereby lowering the risk of depression (42). From this perspective, PA may be understood not only as a form of bodily movement but also as a behavioral context that helps adolescents maintain regular daily routines, gain positive experiences, and enhance psychological vitality (43). In addition, PA may be associated with better sleep and energy levels, reduced fatigue, and more positive emotional experiences. Accordingly, regular participation in PA may be linked to more adaptive physical and psychological functioning, including better stress-coping experiences, more positive emotional states, and fewer DP-related difficulties. Given the cross-sectional design and the absence of biological indicators in the present study, these explanations should be interpreted cautiously and regarded as background evidence rather than directly tested mechanisms (44). Thus, the association between PA and lower levels of DP may be better understood as the combined result of improved physical and psychological states and positive behavioral activation, rather than as the effect of a single pathway. In practical terms, encouraging adolescents to engage in appropriate PA may be a feasible direction for supporting psychological well-being and positive social development.

### 4.2 The mediating role of SCH

The present study found a significant indirect association between PA and DP through SCH, supporting H2 (45). In terms of effect proportion, the SCH pathway made a relatively larger contribution among the three indirect pathways and was stronger than the independent pathway through SCT. This finding suggests that the association between higher PA and lower DP may be partly reflected in adolescents’ stronger connection with the school environment. From the perspective of stage–environment fit theory, PA in school settings is not limited to bodily movement; it also involves social processes such as peer cooperation, teacher–student interaction, rule adherence, and collective participation. These experiences may help strengthen students’ sense of school belonging and psychological membership. Previous studies have often treated SCH as an important predictor of DP (46), whereas other research has focused more on intrapersonal resources, such as self-esteem, self-efficacy, and psychological resilience, in explaining the link between PA and mental health. In contrast, the present study further demonstrates that SCH, as an external contextual resource, showed a statistically significant indirect association between PA and DP and contributed a relatively larger share among the three indirect pathways. This pattern suggests that, among Chinese adolescents, the association between PA and DP may be partly reflected in stronger school connection and greater involvement in the school environment, rather than only in individual psychological characteristics. The current findings further show that SCH may serve as a potential contextual pathway linking PA to DP. This suggests that SCH may help adolescents not only achieve better adjustment but also experience fewer negative emotional problems.

### 4.3 The mediating role of SCT

The present study found a significant indirect association between PA and adolescent DP through SCT, which is consistent with previous research and supports H3 (47). Although the size of this pathway was relatively modest, it still explained a meaningful part of the overall association. This pattern implies that the link between PA and DP is shaped not only by external relational factors, but also by changes in adolescents’ capacity for self-regulation. According to self-regulation theory, SCT is a key psychological resource that enables individuals to maintain goal-directed behavior when facing temptation, stress, and emotional fluctuations, and is therefore central to adaptive development during adolescence (37). PA is inherently characterized by planning, persistence, and rule compliance. Participation in PA often requires individuals to sustain effort, tolerate fatigue, and inhibit impulsive responses, thereby reinforcing self-management and behavioral control through repeated practice (48). In parallel, regular PA may improve executive functioning, strengthen attentional and inhibitory control, and enhance achievement experiences and self-efficacy. Consequently, adolescents may be better able to respond to academic stress, interpersonal difficulties, and negative emotions in constructive ways, rather than showing maladaptive reactions or losing emotional control (49, 50). The present findings further suggest that the association between PA and DP is not solely reflected in a direct pathway, but may also be partly explained by SCT as an internal self-regulatory resource. It is also worth noting that the pathway involving SCT was weaker than the one involving SCH. This pattern suggests that the association between PA and DP may reflect the joint contribution of school-based contextual support and individual self-regulatory capacity, rather than the improvement of a single personal characteristic alone.

### 4.4 The serial mediating roles of SCH and SCT

Further mediation analyses showed that, after SCH and SCT were included in the model, the direct association between PA and DP was no longer statistically significant. In contrast, the serial indirect pathway of “PA → SCH → SCT → DP” was statistically significant and accounted for 21.33% of the total effect, supporting H4 (49). This finding suggests that the association between PA and lower DP in adolescents may be partly reflected through psychosocial pathways involving SCH and SCT, rather than through a purely direct association (51). This pattern is broadly consistent with the social ecological model, which emphasizes that adolescent mental health is shaped by the ongoing interplay between individual characteristics and environmental contexts (52). PA is not only a health-related behavior but also a structured activity embedded in school life (53). Within the school context, PA may provide opportunities for peer interaction, cooperative participation, teacher–student communication, rule adherence, goal persistence, and self-management (21). These experiences may be associated with stronger SCH and better SCT, which in turn are linked to lower DP. In this model, SCH represents an external contextual resource derived from the school environment, whereas SCT reflects adolescents’ internal capacity for self-regulation. The two factors may jointly help explain the indirect association between PA and DP (54). However, given the cross-sectional design, this sequential pathway should not be interpreted as evidence that PA first strengthens SCH, then improves SCT, and finally reduces DP. Alternative explanations, such as SCT influencing SCH or reciprocal associations between SCH and SCT, cannot be ruled out. Therefore, the present findings should be understood as theory-driven statistical associations and should be further tested using longitudinal or intervention designs. Overall, the results provide preliminary evidence that school-based PA, SCH, and SCT may be useful targets for promoting adolescent mental health.

## 5 Limitations and future directions

Several limitations should be considered when interpreting the present findings. First, this study used a cross-sectional design, which limits the ability to determine causal relationships or temporal ordering among PA, SCH, SCT, and DP. Although the serial mediation model of “PA → SCH → SCT → DP” was developed based on self-determination theory and ecological systems theory, it should be understood as a theory-driven statistical pathway rather than direct evidence of causal direction. In addition, the sample was drawn from six schools in the Guangxi Zhuang Autonomous Region, and students may have been influenced by shared school or class-level contexts. However, the present analysis did not use multilevel modeling or cluster-robust standard errors to account for potential clustering. Moreover, alternative mediation structures were not tested, and possible bidirectional associations between SCH and SCT cannot be ruled out. Future studies should consider longitudinal designs, multilevel models, structural equation modeling, or cross-lagged approaches, while incorporating school-level variables to further examine the directionality and robustness of different theoretical models. Second, the study relied mainly on adolescents’ self-reported questionnaires, which may introduce recall bias and common method bias. Although Harman’s one-factor test and single-factor confirmatory factor analysis suggested that common method bias was unlikely to seriously distort the findings, all major variables were collected from the same respondents at the same time point; therefore, this possibility cannot be fully excluded. Future research should combine multiple sources of information, longitudinal follow-up, objective PA measurement, and more rigorous statistical controls to improve the robustness of the findings. Third, although the PA scale used in this study estimated overall PA levels based on exercise intensity, duration, and frequency, the number of items was limited. It may not fully capture the multidimensional characteristics of adolescent PA, such as activity type, exercise context, sedentary behavior, and objectively measured moderate-to-vigorous activity. Future studies could use accelerometers, activity logs, multi-informant reports, or more context-sensitive PA measures to improve measurement precision. Finally, the sample was mainly drawn from selected areas of Guangxi and included a relatively high proportion of junior high school students, whereas senior high school students were less represented. This may limit the generalizability of the findings to adolescents from other regions, school stages, and educational contexts. Although demographic variables such as gender, school stage, residence, and parental education were controlled, the model did not include broader ecological factors, such as family support, peer relationships, school climate, or academic stress. Future research should include more diverse and balanced samples, integrate multilevel ecological variables, and further examine whether different types, intensities, and contexts of PA are differentially associated with SCH, SCT, and DP.

## 6 Conclusion

PA was negatively associated with DP among adolescents. After SCH and SCT were included in the model, the direct association between PA and DP was no longer statistically significant, whereas the indirect pathways through SCH, SCT, and the serial pathway involving both variables were statistically significant. These findings suggest that SCH and SCT may jointly contribute to the association between PA and adolescent DP. Given the cross-sectional design of this study, the results should be interpreted as statistical associations rather than direct evidence of causal relationships.

## Data Availability

The raw data supporting the conclusions of this article will be made available by the authors, without undue reservation.
